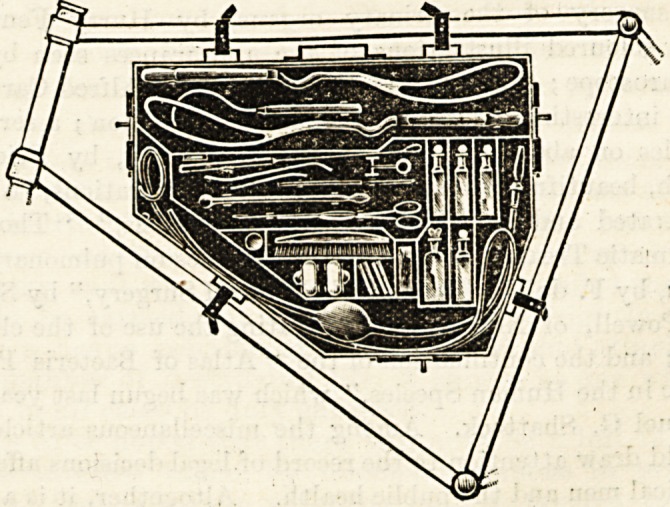# New Appliances and Things Medical

**Published:** 1899-04-22

**Authors:** 


### NEW APPLIANCES AND THINGS MEDICAL.

[We shall be glad to receive, at our Office, 28 & 29, Southampton Street, Strand, London, W.C., from the manufacturers, specimens of all new preparations and appliances which may be brought out from time to time.]

### AN ASEPTIC OBSTETRIC CASE.

(Messrs. Reynolds and Branson, Limited, of Leeds.)

This case has been constructed at the suggestion of Mr. Herbert Rowe, M.R.C.S., Leeds. It is made throughout of block tin, is unlined, and as far as possible has its corners inside rounded off to increase the ease with which the interior may be cleaned. The instruments, &c., are held in position by metal partitions and clips, and if the bottles are removed, the case with its contents *in situ* may be boiled or sterilized in any other convenient way. Its shallowness ($1\frac{7}{16}$ inch) and general shape allows it to be carried on a bicycle, but it is equally conveniently carried in the hand. The list of instruments it contains can be added to or diminished according to requirements. The case illustrated contains five bottles and fourteen instruments (including full-sized forceps and a transfusion apparatus). It is believed that sufficient attention has not hitherto been drawn to the general practitioner's midwifery bag as the probable cause of many cases of septic infection. The ordinary bag remaining in use, as it does, for a long time, and being constantly dipped into by its owner when his hands are in anything but a clean condition, can by no possible effort be rendered properly aseptic.

## Figures and Tables

**Figure f1:**